# Colon Cancer General Knowledge, Attitude and Awareness Channels: A Cross‐Sectional Study

**DOI:** 10.1002/hsr2.70340

**Published:** 2025-04-16

**Authors:** Mu'taz Massad, Mohanad Odeh, Nour Odeh, Leen Abu Sarhan, Rama Alharahsheh, Islam Abu Suilik, Manar ALFaleh, Eman Aladli, Aya Ibrahim, Hashem Abu Serhan

**Affiliations:** ^1^ Department of Internal Medicine, Faculty of Medicine The Hashemite University Zarqa Jordan; ^2^ Department of Clinical Pharmacy and Pharmacy Practice, Faculty of Pharmaceutical Sciences The Hashemite University Zarqa Jordan; ^3^ Department of Ophthalmology Hamad Medical Corporations Doha Qatar

**Keywords:** attitude, colon cancer, colonoscopy, knowledge, screening

## Abstract

**Background:**

Colon cancer is one of the most widespread cancers in Jordan. Screening of colon cancer aids in reducing its incidence and mortality rates. Awareness of colon cancer and its screening tools has a fundamental role in increasing screening participation. The information about Jordanians' awareness of colon cancer screening is inadequate.

**Objective:**

This study aims to assess the Jordanian population's level of awareness about colon cancer, including basic knowledge, screening tools, attitudes toward early screening, and preferred methods for spreading awareness.

**Methods:**

This is an analytical cross‐sectional study. The study was conducted using both online and paper‐based validated, and reliable questionnaires which were distributed throughout the entire community. Knowledge scores (KS range −10 to +10) and attitude scores (AS range −8 to +8) were calculated. Univariate analysis and logistic regression model were carried out. The nominal‐by‐nominal strength was also calculated.

**Results:**

Information was collected from 1050 participants aged 18 to 70 years. with 63.6% being female responders. Participants with negative knowledge scores ‘'KS ≤ zero” were greater than good knowledge scores ‘'KS > 4 out of 10” (25.8% vs 11.4%). age, gender, insurance, working in the medical field, education, monthly income, smoking, and family history of cancer showed statistically significant associations with KS (*p* < 0.005, Cramer's *V* > 0.1). The strongest predictor for KS was the level of education (the postgraduate group showed OR = 4.64, *p* = 0.001, 95% CI = 1.96–11.0). Most participants (87.6%) had a positive attitude toward screening (AS ≥ 1). There were no associations between knowledge and attitude scores (*p* > 0.05). Unlike newspapers, social media was perceived as the most effective (70%) way to education.

**Conclusion:**

One‐quarter of the population is in crucial need for proper education, especially among young groups. This study forms a good basis and provides a solid foundation for health authorities to implement the necessary measures to address this issue.

## 1 Introduction

Colorectal cancer (CRC) is a serious health problem, regarded as the third most common cancer in males and the second in females worldwide [1]. In Jordan, a study conducted on CRC in 2020 found that CRC is the second‐leading cancer and the third deadly malignancy [2]. The study recorded 11,559 new cancer cases and 6190 cancer deaths [2].

The burden of CRC is predicted to keep rising due to the high prevalence of related risk factors among Jordanians [3]. Screening techniques can dramatically lower CRC incidence and mortality rates [4]. A systemic review shows that colonoscopy, a screening method, reduces about 69% of CRC incidence and 68% of CRC mortality [5].

The financial burden that colon cancer places on the national budget poses another challenge. In the United States, the average annual healthcare expenditure for working‐age cancer survivors is three times higher than for the same population without a history of cancer [6]. Furthermore, one study showed that cancer care costs are increasing two to three times faster than other healthcare costs [6]. This put a greater economic burden on the state budget, particularly in Jordan, a middle‐income country where the government covers the cost of cancer treatment [7].

Screening means detecting a disease when there are no symptoms [8]. Colon cancer screening is essential for its prevention and early detection. The majority of CRCs are caused by pre‐existing polyps [9]. Detecting these polyps early makes the disease highly curable by treating them before they progress into cancer [10]. Furthermore, survival and recurrence rates depend on the cancer stage at the time of diagnosis [11, 12]. One study found that the 5‐year survival rate for CRC drops from 90% to 10% when it progresses from the localized stage into the advanced stage [11].

In many countries, the number of CRC cases is increasing year after year [13]. The need for screening program adoption becomes extremely critical. However, only a small percentage of the target population worldwide is offered CRC screening [14]. In Jordan, despite cancer's high prevalence, the national health authorities have not yet established a clear plan or guidelines for CRC screening [14].

A successful screening program requires a high participation rate. Numerous factors can affect an individual's decision to have a screening, including their age, gender, marital status, health insurance, test worries, and background knowledge [15, 16, 17, 18]. People are more willing to undergo screening if they know about the illness and its screening options [19]. Therefore, enhancing awareness of colon cancer is essential before implementing screening programs to increase participation [20]. This study aims to evaluate the level of awareness among the Jordanian population regarding basic knowledge of colon cancer and its screening tools, assess their attitudes toward early screening, and identify their preferred methods for raising awareness, to guide healthcare providers and policymakers in planning CRC screening programs. The researchers in this study hypothesize that the Jordanian population will exhibit a lack of awareness about colon cancer and its screening methods. consequently, this will directly influence their attitudes toward colon cancer screening programs.

## 2 Methods

### 2.1 Study Design

In this analytical cross‐sectional study, data was collected through a self‐completion questionnaire. Some questions have been taken from a previous research questionnaire [21, 22], and the rest were designed by the researchers based on research objectives. The study aims to assess and analyze the level of awareness of colon cancer screening and the attitude toward it. The questionnaire includes 25 multiple‐choice questions and 2 Likert scale questions written in two sheets that need 10–15 min to answer. The survey consists of three sections: the demographic, the knowledge, and the attitudes toward colon cancer screening sections.

The socio‐demographic section includes age, gender, residency, nationality, monthly income, health insurance, educational level, smoking status, and chronic illnesses. The questionnaire also inquiries about personal and family history of colon cancer, family history of cancerous disease, and previous experience with colon cancer screening. The second section asks whether the participant had previously heard about colon cancer and its screening. The rest of the questions assess the general knowledge about colon cancer including the definition of colon, level of colon cancer prevalence, its relations to endometrial and urinary tract cancers, as well as the age of screening and its interval.

Participants' perceptions of the role of screening in early detection and decreasing progression of colon cancer were obtained. Additionally, they asked whether they think that early detection helps in the recovery of cancer since that perception may affect their attitude toward screening. The third section aims to assess the community's readiness to receive a national program for colon cancer screening by inquiring about the acceptance of colonoscopy and the willingness of the population to commit to a colon cancer screening program. Finally, given the importance of the medical sector's role in promoting awareness, the questionnaire concluded by asking the participants about the most attractive education method from their point of view. The questions have been formulated in Arabic in a simplified manner and easy‐to‐understand terms for everyone. It was reviewed by a specialist in gastroenterology, hepatology, and endoscopy. Validity and reliability were confirmed. The questionnaire was designed using the KAP format.

### 2.2 Sample Size

The 2023 census showed Jordan's population to be 10,488,014 based on the last United Nations data [23]. Accordingly, the minimum sample size was determined to be 385, as calculated using a sample size calculator [24]. The confidence level was considered 95% and the margin of error was 5%.

### 2.3 Survey Validity and Reliability

Content validity was assessed using the content validity ratio (CVR) as proposed by Lawshe [25]. To establish content validity, eight subject matter experts reviewed the suggested questions in the survey. Each expert evaluated whether each item was essential, useful but not essential, or not necessary. The CVR for each item was then calculated using the formula:

$$\text{CVR}\;=\left(n_{e}\;-N/2\right)/N/2$$

where *n*_e_ is the number of experts who rated the item as essential, and N is the total number of experts. Only items with a CVR greater than 0.741 and a *p*‐value less than 0.05 were retained, ensuring that the included items were deemed essential by a significant proportion of experts.

Reliability of the questionnaire was evaluated through the assessment of internal consistency. Cronbach's alpha, a measure of overall reliability, was calculated for the knowledge and attitude sections in the questionnaire. The resulting Cronbach's alpha was 0.82, indicating a high level of internal consistency.

### 2.4 Data Collection

A total of 1065 questionnaires were collected, and 15 invalid samples were excluded. Inclusion criteria: Participants between 18 and 70 who can complete the questionnaire mentally and physically. Missing one or more answers was deemed invalid.

Data were collected through soft and hard copy questionnaires, which were done by fifth‐year medical students from Hashemite University. The team consists of seven students and a specialist in gastroenterology, hepatology, and endoscopy. The sample was recruited during January and February of 2023.

56.3% [26] of the Jordanian population is concentrated in Amman and Al‐Zarqa, and therefore the biggest part of the sample was collected in the streets, shops, and gathering places in those two governorates to ensure that the sample is more reliable. Other governorates were included in the online questionnaire published in social media groups.

Students ensured that the participants were comfortable, not in a hurry, and mentally prepared to participate, to gather more reliable information and increase their willingness and acceptance to fill out the questionnaire carefully. Students read the questions for illiterates and filled them in according to the participants' answers without affecting their choice. After receiving the questionnaire, students verified that the answers were complete and that the questionnaire was usable.

Ethical approval was obtained from the Institutional Review Board at Hashemite University (HU). Verbal consent was obtained from the participants, and they had the right to refuse to fill it out. The identity of the participants has remained anonymous.

#### 2.4.1 Knowledge Score

Each correct answer of the nine questions scored with +1 mark, each wrong answer scored with −1 mark, and the answer with do not know has a 0 mark. The sum up of total marks was the total score.

#### 2.4.2 Knowledge Score Categories

Negative knowledge if the total score = less than 0, the category do not know was assigned to those who had a total score = 0, having a score between 1 and 4 out of 9 was categorized as a knowledgeable group, and a score above 4 was categorized as having good knowledge. For the purpose of binary logistic regression, the binary groups were: a group with a total score equal to zero or less (group who needs education), a group with a total score equal to or more than 1 (the group with knowledge).

#### 2.4.3 Attitude Score

The following marks were assigned for each answer: Agree (+1), Strongly agree (+2), Disagree (−1), Strongly disagree (−2), Neutral (0). The range for the scores was from −8 to +8.

#### 2.4.4 Attitude Score Categories

A negative attitude was assigned to those who had an attitude score of zero or less, while a positive attitude was assigned to those with scores equal or more than 1.

### 2.5 Data Synthesis (Statistical Analysis)

Data were coded and transferred to SPSS for Windows, version 25, for statistical analysis (SPSS Inc, Chicago, IL, USA). For data analysis, descriptive analysis was first conducted to present participant characteristics. The chi‐squared test (*X* ^2^) for association between variables and level of knowledge (or level of attitude) was used (Fisher's exact test was used in cases where the expected count was less than 5). To measure the strength of the association of a nominal‐by‐nominal relationship (which is a measure of effect size), the Cramer's V coefficient factor was also calculated and illustrated, with a strength of association value of 1 indicating complete association, “0” indicating no association, > 0.25 indicating a very strong relationship, > 0.15 indicating a strong relationship, > 0.1 indicating a moderate relationship, and > 0.05 indicating a weak relationship [27].

The degrees of freedom for the chi‐square between practices were illustrated using the formula *df* = (*r* − 1)(*c* − 1), where *r* is the number of categories within the demographic variable and *c* is the number of options related to item options. For all tests in the present study, a *p*‐value (two‐sided) of < 0.05 is considered to indicate a statistically significant difference [28].

To ascertain the effects of different variables on levels of knowledge, a binomial logistic regression was performed. A backward stepwise approach was utilized to eliminate independent predictors that did not contribute significantly, thereby optimizing model performance. The cutoff points for stepwise entry were set at a probability of 0.05. Model significance was assessed using the Omnibus Tests of Model Coefficients. Additionally, the Nagelkerke R² was used as a measure of goodness of fit and to explain the variance in knowledge scores, and the percentage accuracy in classification was reported. The odds ratio along with the 95% confidence intervals were also reported.

## 3 Results

A total of 1050 participants were included. Most were females (63.6%), Jordanian (96.7%), nonmedical field workers (91.0%), and non‐smokers (68.8%). Over half had insurance (65.2%). Regarding monthly income, most earned between 300 and 699 (34.2%), followed by those with no monthly income (30.0%). Regarding educational level (64.7%) had a bachelor's degree. (59.4%) of participants were living in Amman‐Jordan followed by Al Zarqa governorate (19.7%) and the majority had no chronic illnesses (81.6%) (Table 1).

**Table 1:** Demographic characteristics.

Variable	N (%)
Gender	
Female	668 (63.6)
Male	382 (36.4)
Insurance	
Yes	685 (65.2)
No	365 (34.8)
Nationality	
Jordanian	1015 (96.7)
Not Jordanian	35 (3.3)
Monthly income	
No Monthly Income	315 (30.0)
Less than 300JD	203 (19.3)
300‐699 JD	359 (34.2)
700‐1000 JD	95 (9.0)
More than 1000 JD	78 (7.4)
Medical field worker	
Yes	94 (9.0)
No	956 (91.0)
Educational level	
Less than Secondary	71 (6.8)
Secondary	197 (18.8)
bachelor's degree	679 (64.7)
postgraduate education	103 (9.8)
Smoking	
Yes	237 (22.6)
Sometimes	91 (8.7)
No	722 (68.8)
Chronic illnesses	
Yes	193 (18.4)
No	857 (81.6)
residency	
Irbid	72 (6.9)
Balqa	23 (2.2)
Zarqa	207 (19.7)
Tafila	10 (1.0)
Aqaba	12 (1.1)
Karak	16 (1.5)
Mafraq	27 (2.6)
Jerash	8 (0.8)
Ajloun	14 (1.3)
Aman	624 (59.4)
Madaba	22 (2.1)
Ma'an	15 (1.4)

The provided data shows that most participants had neither a personal history (98.3%) nor a family history (76.9%) of colon cancer, and half of them had no family history of cancerous diseases (50%). (68.6%) had heard about colon cancer, and only (35.8%) had heard about its early detection. (96.3%) had not undergone a colon cancer screening test before. The majority (91.7%) thought that there is not enough awareness of colon cancer in their residential area. (Table 2)

**Table 2:** Descriptive data about colon cancer.

Variable	N (%)
Family history of cancer	
No	525 (50)
Yes, 1st relative	280 (26.6)
Yes, 2nd relative	245 (23.4)
Family history of colon cancer	
No	807 (76.9)
Yes, 1st relative	130 (12.4)
Yes, 2nd relative	113 (10.8)
Hearing about colon cancer	
No	330 (31.4)
Yes	720 (68.6)
Hearing about the early detection of colon cancer	
No	674 (64.2)
Yes	376 (35.8)
Do you think there is enough awareness of colon cancer in your residential area	
No	963 (91.7)
Yes	87 (8.3)

The data shows participants' background knowledge about colon, colon cancer, and it is screening. (59.3%) knew that the colon is part of the large intestine while (17.9%) did not know what the colon is. (42.7%) do not know what is the colon cancer prevalence in Jordan and (20.2%) think that it's among the three most common cancers. Most participants were unaware of the recommended age for colon cancer screening and the screening interval (49.9% and 56.9%, respectively). Only 6.6% knew screening starts at age 50, and 4.3% knew the screening interval is 5 to 10 years. 73.9% believed colonoscopy could help detect colon cancer. Additionally, 88.7% thought early detection could reduce disease progression, and 67.8% believed it could be cured if detected early. Finally, 58% and 44% did not know whether colon cancer is related to endometrial and urinary tract cancers, respectively (Table 3).

**Table 3:** Colon cancer screening knowledge.

Variable	N (%)
Colon is	
Part of small intestine	116 (11.0)
Part of large intestine	623 (59.3)
Part of stomach	123 (11.7)
Don't know	188 (17.9)
Colon Cancer Prevalence in Jordan	
Don't know	448 (42.7)
Medium (out of 5–10 most popular)	327 (31.1)
Widespread (among the three most common cancer)	212 (20.2)
Rare (among the least common cancer)	63 (6.0)
Age of colon cancer screening	
At the age of 20 years	102 (9.7)
At the age of 40 years	169 (16.1)
At the age of 50 years	69 (6.6)
At the age of 70 years	3 (0.3)
When symptoms appear	183 (17.4)
Don't know	524 (49.9)
Periodic colon cancer screening interval	
Don't know	597 (56.9)
six months to one year	253 (24.1)
One year to three years	140 (13.3)
Five years to ten years	45 (4.3)
Ten years to fifteen years	15 (1.4)
Do you think that a colonoscopy can help detect colon cancer?	
Do not know	241 (23.0)
No	33 (3.1)
Yes	776 (73.9)
Do you think that early detection of colon cancer has a role in reducing the progression of the disease?	
Do not know	100 (9.5)
No	19 (1.8)
Yes	931 (88.7)
Can colon cancer be cured when detected early?	
Do not know	297 (28.3)
No	41 (3.9)
Yes	712 (67.8)
Do you think there is a relationship between colon cancer and endometrial cancer?	
Do not know	609 (58.0)
No	240 (22.9)
Yes	201 (19.1)
Do you think there is a relationship between colon cancer and urinary tract cancer?	
Do not know	462 (44.0)
No	176 (16.8)
Yes	412 (93.2)

### 3.1 Knowledge Score

The mean knowledge score was 1.88 (SD = 2.2) out of 9. Almost two‐thirds of the participants (62.8%) had a knowledge score between 1 and 4 out of 10 (knowledgeable), and participants with negative knowledge (less than 0) were 12.5% of the recruited sample, which was more than those with good knowledge (11.4%). Those who would need awareness intervention were almost one‐quarter of the participants (25.8%), which includes participants with scores less than zero (12.5%) or equal to zero (13.3%) (Supporting Information S1: Table 1).

### 3.2 Knowledge Score Association With Variables and Strength of the Relationship

Table 4 shows the association and the strength of the relationship as measured by Cramer's V factor. Most of the variables demonstrated statistically significant association with the level of knowledge except for nationality, claiming a previous awareness about colon cancer and colon cancer early detection. The strongest association was with education level (*X* ^2^ = 299.3, *p* < 0.001, Cramer's *V* = 0.53) followed by Gender (*X* ^2^ = 52.1, *p* < 0.001, Cramer's *V* = 0.22) Smoking (*X* ^2^ = 29.8, *p* < 0.001, Cramer's *V* = 0.17) and age (*X* ^2^ = 45.8, *p* < 0.001, Cramer's *V* = 0.15).

**Table 4:** Knowledge score association with variables and strength of the relationship.

Variable	Negative Knowledge	Do not know	Knowledge	Good Knowledge	Total	X^2^ a	P^b^	Cramer's V
Gender								
Female	91 (13.5%)	83 (12.3%)	456 (67.8%)	43 (6.4%)	673 (100%)	52.1	< 0.001	0.22
Male	40 (10.6%)	57 (15.1%)	203 (53.8%)	77 (20.4%)	377 (100%)			
Insurance								
No	33 (9.1%)	34 (9.3%)	250 (68.7%)	47 (12.9%)	364 (100%)	16.1	0.001	0.12
Yes	73 (10.6%)	106 (15.5%)	409 (59.6%)	98 (14.3%)	686 (100%)			
Nationality								
Jordanian	130 (12.8%)	137 (13.5%)	634 (62.4%)	115 (11.3%)	1016 (100%)	4.1	0.25	0.06
Not Jordanian	1 (2.9%)	3 (8.8%)	25 (73.5%)	5 (14.7%)	34 (100%)			
Monthly income								
No Monthly Income	39 (12.1%)	34 (10.5%)	231 (71.5%)	19 (5.9%)	323 (100%)	63.9	< 0.001	0.14
Less than 300JD	15 (7.6%)	24 (12.1%)	116 (58.6%)	43 (21.7%)	198 (100%)			
300‐699 JD	48 (13.4%)	50 (14.0%)	205 (57.4%)	54 (15.1%)	357 (100%)			
700‐1000 JD	17 (18.1%)	15 (16.0%)	59 (62.8%)	3 (3.2%)	94 (100%)			
More than 1000 JD	12 (15.4%)	17 (21.8%)	48 (61.5%)	1 (1.3%)	78 (100%)			
Medical field worker								
No	111 (11.6%)	127 (13.3%)	601 (62.9%)	117 (12.2%)	956 (100%)	12.6	0.006	0.11
Yes	3 (3.2%)	13 (13.8%)	58 (61.7%)	20 (21.3%)	94 (100%)			
Educational level								
Less than Secondary	32 (47.8%)	4 (6.0%)	28 (41.8%)	3 (4.5%)	67 (100%)	299.3	< 0.001	0.53
Secondary	19 (9.2%)	21 (10.2%)	139 (67.5%)	27 (13.1%)	206 (100%)			
bachelor's degree	39 (6.0%)	95 (14.5%)	446 (68.3%)	73 (11.2%)	653 (100%)			
postgraduate education	3 (2.9%)	20 (19.6%)	65 (63.7%)	14 (13.7%)	102 (100%)			
Smoking								
Sometimes	5 (5.5%)	8 (8.8%)	63 (69.2%)	15 (16.5%)	91 (100%)	29.8	< 0.001	0.17
No	61 (8.4%)	101 (13.8%)	465 (63.7%)	103 (14.1%)	730 (100%)			
Yes	23 (10.0%)	44 (19.2%)	131 (57.2%)	31 (13.5%)	229 (100%)			
Presence of Chronic illnesses								
No	112 (13.0%)	114 (13.3%)	549 (63.8%)	85 (9.9%)	860 (100%)	11.9	0.008	0.11
Yes	19 (10.0%)	26 (13.7%)	110 (57.9%)	35 (18.4%)	190 (100%)			
Family history with cancer in general								
No	75 (14.3%)	73 (13.9%)	318 (60.6%)	59 (11.2%)	525 (100%)	14.3	0.026	0.08
Yes, 1st line relatives	34 (12.3%)	30 (10.9%)	170 (61.6%)	42 (15.2%)	276 (100%)			
Yes 2nd line relatives	22 (8.8%)	37 (14.9%)	171 (68.7%)	19 (7.6%)	249 (100%)			
Family history with colon cancer								
No	113 (14.0%)	116 (14.4%)	490 (60.8%)	87 (10.8%)	806 (100%)	20.3	0.002	0.1
Yes, 1st line relatives	13 (9.9%)	10 (7.6%)	85 (64.9%)	23 (17.6%)	131 (100%)			
Yes 2nd line relatives	5 (4.4%)	14 (12.4%)	84 (74.3%)	10 (8.8%)	113 (100%)			
Hearing about colon cancer								
No	44 (13.3%)	36 (10.8%)	203 (61.1%)	49 (14.8%)	332 (100%)	7.4	0.06	0.09
Yes	87 (12.1%)	104 (14.5%)	456 (63.5%)	71 (9.9%)	718 (100%)			
Hearing about the early detection of colon cancer								
No	86 (12.8%)	81 (12.0%)	427 (63.4%)	80 (11.9%)	674 (100%)	2.9	0.93	0.05
Yes	45 (12.0%)	59 (15.7%)	232 (61.7%)	40 (10.6%)	376 (100%)			
Age								
Age 18 to 25	40 (10.7%)	43 (11.5%)	276 (73.8%)	15 (4.0%)	374 (100%)	45.8	< 0.001	0.15
Age 26 to 40	44 (13.8%)	39 (12.3%)	180 (56.6%)	55 (17.3%)	318 (100%)			
Age more than 40	47 (13.1%)	58 (16.2%)	203 (56.7%)	50 (14.0%)	358 (100%)			

^a^ *X* ^2^, chi‐squared test.

^b^ Significant level at *p* = 0.05 two‐sided.

More than three‐quarters of respondents (36%: strongly agree, 41%: agree) are agreed to accept colonoscopy as a screening method. Additionally (81.1%) (37%: strongly agree,44.1%: agree) agree to adhere to the periodic colon cancer screening if advised by. Half of the respondents (20.1% strongly agree, 33.1% agree) are willing to pay out‐of‐pocket for colon cancer screening if health insurance does not cover it, while the rest either disagree or are neutral. Finally, the majority support adopting colon cancer screening as a national program in Jordan (48.3% strongly agree, 37.8% agree) (Table 5).

**Table 5:** Participants' attitude toward colon cancer screening.

Variable	N (%)
Acceptance of colonoscopy to detect colon cancer	
Agree	433 (41.2)
Strongly agree	378 (36.0)
Disagree	50 (4.8)
Strongly disagree	24 (2.3)
neutral	165 (15.7)
acceptance to adhere the periodic colon cancer screening, if doctor advises that	
Agree	463 (44.1)
Strongly agree	388 (37.0)
Disagree	42 (4.0)
Strongly disagree	16 (1.5)
neutral	141 (13.4)
agreement to conduct the early screening for colon cancer on personal account if there's no health insurance	
Agree	348 (33.1)
Strongly agree	211 (20.1)
Disagree	178 (17.0)
Strongly disagree	45 (4.3)
neutral	268 (25.5)
level of support for inclusion of early screening for colon cancer in the national program	
Agree	397 (37.8)
Strongly agree	507 (48.3)
Disagree	29 (2.8)
Strongly disagree	15 (1.4)
neutral	102 (9.7)

### 3.3 Attitude Score and Its Association With Knowledge Score

Most of the participants (87.6%) had a positive attitude score ≥ 1 toward colon cancer screening. Despite the level of knowledge most of the participants demonstrated a positive attitude, but there was no statistically significant association between the level of knowledge and the attitude (*X* ^2^ = 0.73, Cramer's *V* = 0.026, *p* = 0.86) (Table 6 and Supporting Information S1: Table 2).

**Table 6:** Attitude score and its association with knowledge score.

		Attitude category		Total			
		Negative	Positive		*X* ^2^ a	P^b^	Cramer's V
Negative Knowledge	Count	15 (11.5%)	116 (88.5%)	131 (100%)	0.73	0.86	0.026
Do not know	Count	19 (13.6%)	121 (86.4%)	140 (100%)			
Knowledge	Count	79 (12.0%)	580 (88.0%)	659 (100%)			
Good Knowledge	Count	17 (14.2%)	103 (85.8%)	120 (100%)			

^a^ *X* ^2^, chi‐squared test.

^b^ Significant level at *p* = 0.05 two‐sided.

### 3.4 Binary Logistic Regression Model to Identify Factors That Impact the Knowledge Score

Six models of stepwise backward logistic regression were created to identify the impact of independent variables on the knowledge score, nonsignificant independent predictors were removed. Table 7 shows the final model, which was statistically significant (*p* < 0.001), and explained 34.5% (Nagelkerke *R* ^2^) of the variance in knowledge score and correctly classified 74.2% of cases. All variables in the table demonstrated an independent statistically significant impact on predicting knowledge score (Age, Insurance, working in the medical field, education, and smoking). Nevertheless, the strongest impact was the level of education – postgraduate group (OR = 4.64, *p* = 0.001, 95% CI = 1.96–11.0), i.e., an increase in one unit (i.e., being postgraduate) increases the odds of having higher knowledge score by 4.64. followed by being a nonsmoker with odds of 2.36 (OR = 2.36, *p* = 0.011, 95%CI = 1.2–4.6) (Table 7).

**Table 7:** Binary logistic regression model to identify factors that impact the knowledge score.

Variables a	Categories	Significant	Odds Ratio	95% Confidence Interval for Odds Ratio	
				Lower	Upper
Age	Age Three groups	0.002			
	Age 18 to 25	Reference			
	Age 26 to 40	0.001	1.853	1.307	2.628
	Age more than 40	0.098	1.329	0.949	1.863
Insurance	No	Reference			
	Yes	0.001	1.714	1.243	2.364
Medical Field worker	No	Reference			
	Yes	0.004	1.779	1.202	2.633
Education	Education Four groups	0.001			
	Less than Secondary	Reference			
	Secondary	0.008	1.992	1.202	3.303
	bachelor's degree	0.253	1.260	0.847	1.875
	postgraduate education	0.001	4.640	1.955	11.013
Smoking	Smoking Three groups	0.026			
	Sometimes	Reference			
	No	0.011	2.363	1.219	4.580
	Yes	0.930	1.015	0.729	1.413

^a^ a. The variables listed in the table are those that significantly impact the knowledge score across all models generated by the backward regression method. Six models were created in total. The variables included in the first model were: Age, Insurance, Working in the medical field, Education, and Smoking. Additional variables considered were Gender, Nationality, Monthly income, Family history of cancer, Family history of colon cancer, Having a chronic disease, Hearing about colon cancer, Hearing about early detection of colon cancer, and Attitude score.

Regarding the methods that could be used in raising awareness, the participants think that social media (70%), advertising campaigns (46.7%), information from the medical field (46.3%), television (41%), awareness seminars/lectures (35%), and paper publications with attractive designs (35%) are all very effective in descending order. Social media recorded the highest percentage of effectiveness at (70%) compared to other methods, While newspapers recorded the highest percentage of ineffectiveness at (32.2%) (Supporting Information S1: Table 3).

## 4 Discussion

This study emphasizes measuring the degree of knowledge about colon cancer screening on a wide sociodemographic basis in Jordan. The result shows that about 12.75% of the recruited sample had negative knowledge about colon cancer and its screening, which is more than those with good knowledge (11.4%). People who had no knowledge were 13.3% of the recruited sample, so about one‐quarter of the participants, who had negative knowledge or no knowledge, need education about colon cancer screening. 62.8% (almost two‐thirds of the sample) were classified as knowledgeable (having scores between 1 and 4 out of 10), it's a reassuring percentage as dealing with this group with the intention to enhance their knowledge about colon cancer screening is much easier than if they have negative or no knowledge.

Furthermore, the study highlights that almost one‐quarter of the population (25.8%) lacks basic knowledge of colon cancer and its screening programs. In contrast, another cross‐sectional study was conducted in 2010 to investigate the knowledge and beliefs of Jordanians toward CRC screening among 150 participants whose ages were 50 and above. The study found Only 26% and 22% of the participants had previous basic knowledge about colon cancer and colon cancer screening respectively [29]. This discrepancy between the results of the two studies might be attributed to our larger and more targeted sample. Additionally, the significant increase in access to information through the World Wide Web and social media has likely contributed to growing awareness and knowledge about colon cancer screening over time.

Focusing on the attitude of the participants toward colon cancer screening, there is no significant relation between a positive attitude and good knowledge. Despite varied knowledge levels, most of the participants 87% had a positive attitude regardless of their background knowledge about colon cancer screening. 81.8% of the recruited sample agreed to adhere to the periodic colon cancer screening if their doctor advises that and half of the sample even approved conducting the early colon cancer screening by self‐payment if they have no health insurance. These findings reinforce the results of a study about CRC prevention and care in Jordan, which collected a stratified random sample of 3196 participants from all 12 governments of Jordan, 69% had a positive attitude towards CRC screening performance in the future [30]. This means if the population is educated about colon cancer, they will be willing to enroll in colon cancer screening, hence it's a good motive for the decision‐makers to take action to increase the awareness of the population and to implement an organized colon cancer screening program.

Additionally, the study indicates and we notice that the level of education is a significant factor in determining the level of knowledge about colon cancer and colon cancer screening methods.

This study highlights that as the level of education increases, so does the percentage of participants with accurate knowledge about colon cancer and its screening methods, while the percentage of those with negative knowledge nearly disappears. (i.e., negative knowledge was present in around half of the participants among “less than secondary” and nearly zero among “postgraduate”). The level of knowledge is also impacted by gender, followed by smoking, and then age where it was observed to be higher among females, people aged 18–25, and those who occasionally smoke.

Education remains crucial regardless of nationality, monthly income, family history of cancer, family history of colon cancer, having a chronic disease, hearing about colon cancer, and hearing about early detection of colon cancer, where all the percentages of Knowledgeable people in these categories are close.

This contradicts the cross‐sectional study that was carried out in 2019, which involved six hundred participants and revealed that there was no significant association between knowledge of CRC screening methods and gender, age, occupation, or educational level [31]. Furthermore, it demonstrated that the most important factor affecting knowledge about early detection methods of colon cancer was complaining of symptoms that raised suspicion about cancer, followed by having a positive family history of CRC [31]. Education is also crucially needed for younger individuals, smokers, and nonmedical field workers.

People with negative knowledge under the age of 40 have approximately twice the percentage of older people. Specifically, it was 10.7% among people aged 18–25, 13.8% among people aged 25–40, and 13.1% over 40. Furthermore, the results show that the percentage of people with negative knowledge among smokers (whether they were regular (10%) or occasional (5.5%) smokers) is nearly double the percentage among nonsmokers (8.4%). About a quarter of people who are not medical field workers have negative knowledge and donot know about colon cancer screening methods, compared to around 15% of those who are.

Adequate knowledge about colon cancer screening is crucial for early diagnosis and better disease prognosis. Effective educational methods are needed to ensure this knowledge. Our results indicate that 70% of participants believe social media is the most effective method for disseminating information, whereas newspapers are seen as less effective. Therefore, leveraging social media to spread awareness rapidly can significantly enhance public knowledge.

Similar to all self‐reported analytical studies, one significant limitation is the potential for response and recall bias. Self‐reported surveys rely on participants' subjective perceptions, which can be influenced by their emotions, beliefs, or personal biases. To counteract such limitations, we recruited a relatively large sample size with validity and reliability measures for the survey. Despite standard limitations, the findings of this study can be used to plan future comprehensive research before creating educational content and organizing training‐based interventions to further advance the implementation of the CRC screening in Jordan.

## 5 Conclusion

The findings suggest that most of the population is knowledgeable about colon cancer and its screening. Despite that, nearly one‐quarter of the population requires substantial education on colon cancer. A positive attitude towards colon cancer was not necessarily linked with knowledge. Furthermore, social media was identified as the most effective channel for spreading awareness in contrast to newspapers. These results highlight the importance of establishing health education programs to increase awareness. Consequently, this can enhance attitudes toward screening programs, particularly among vulnerable populations. Policymakers are encouraged to develop interventions to achieve this goal.

## Author Contributions

**Mu'taz Massad:** conceptualization, investigation, methodology, validation, software, resources. **Mohanad Odeh:** conceptualization, investigation, funding acquisition, formal analysis, software, supervision. **Nour Odeh:** writing–original draft, validation, visualization, formal analysis. **Leen Abu Sarhan:** validation, visualization, investigation, formal analysis, project administration. **Rama Alharahsheh:** investigation, validation, formal analysis, project administration. **Islam Abu Suilik:** investigation, funding acquisition, validation, software. **Manar ALFaleh:** investigation, validation, formal analysis, data curation. **Eman Aladli:** investigation, validation, data curation, software. **Aya Ibrahim:** investigation, validation, software. **Hashem Abu Serhan:** conceptualization, writing–review and editing, funding acquisition.

## Ethics Statement

Ethical approval was obtained from the Institutional Review Board at Hashemite University (HU).

## Consent

Verbal consent was obtained from the participants, and they had the right to refuse to fill it out. The identity of the participants has remained anonymous.

## Conflicts of Interest

The authors declare no conflicts of interest.

## Transparency Statement

The lead author Hashem Abu Serhan affirms that this manuscript is an honest, accurate, and transparent account of the study being reported; that no important aspects of the study have been omitted; and that any discrepancies from the study as planned (and, if relevant, registered) have been explained.

## Supporting information

Supporting information.

## Data Availability

Data sharing is not applicable to this article as no new data were created or analyzed in this study. All data generated or analyzed during this study are included in this article. Further enquiries can be directed to the corresponding author.
