# Supplementary material for: Colon Cancer General Knowledge, Attitude and Awareness Channels: A Cross‐Sectional Study
Source: Health Sci Rep. 2025 Apr 16;8(4):e70340. doi: 10.1002/hsr2.70340 (PMC12003555; doi:10.1002/hsr2.70340)
Supplement: Supplementary file 1 — Supporting information. [file HSR2-8-e70340-s001.docx]

**Supplementary Table 1.** Knowledge score of participants.

| Knowledge Scores | Frequency |
| --- | --- |
| Negative Knowledge (<0) | 131 (12.5) |
| Do not know (Score 0) | 140 (13.3) |
| Knowledge (1-4) | 659 (62.8) |
| Good Knowledge (5-9) | 120 (11.4) |
| Total | 1050 (100.0) |

**Supplementary Table-2.** Attitude scores of participants.

| Attitude score | Frequency (%) |
| --- | --- |
| Negative (<0) | 130 (12.4) |
| Positive (>1) | 920 (87.6) |
| Total | 1050 (100) |

**Supplementary Table 3.** The effectiveness of different methods in raising awareness about the early detection of colon cancer from the perspective of the participants

| Variable | N (%) | |
| --- | --- | --- |
| social media | |  |
| ineffective | 64 (6.1) | |
| effective | 184 (17.5) | |
| Very effective | 735 (70.0) | |
| Medium | 67 (6.4) | |
| television | |  |
| ineffective | 103 (9.8) | |
| effective | 290 (27.6) | |
| Very effective | 431 (41.0) | |
| Medium | 226 (21.5) | |
| advertising campaigns | |  |
| ineffective | 94 (9.0) | |
| effective | 273 (26.0) | |
| Very effective | 490 (46.7) | |
| Medium | 193 (18.4) | |
| awareness seminars/ lectures | |  |
| ineffective | 109 (16.9) | |
| effective | 253 (23.7) | |
| Very effective | 456 (35.0) | |
| Medium | 232 (24.4) | |
| Paper publications with attractive designs | |  |
| ineffective | 177 (16.9) | |
| effective | 249 (23.7) | |
| Very effective | 368 (35.0) | |
| Medium | 256 (24.4) | |
| Information from the medical field | |  |
| ineffective | 86 (8.2) | |
| effective | 312 (29.7) | |
| Very effective | 486 (46.3) | |
| Medium | 166 (15.8) | |
| newspaper | |  |
| ineffective | 338 (32.2) | |
| effective | 166 (15.8) | |
| Very effective | 244 (23.2) | |
| Medium | 302 (28.8) | |
